# Supplementary material for: Identification of EcpK, a bacterial tyrosine pseudokinase important for exopolysaccharide biosynthesis in Myxococcus xanthus
Source: J Bacteriol. 2025 Mar 11;207(4):e00499-24. doi: 10.1128/jb.00499-24 (PMC12004946; doi:10.1128/jb.00499-24)
Supplement: Supplemental material — Figures S1 to S8, Tables S1 and S2, and supplemental references. [file jb.00499-24-s0001.pdf]

Supporting Information

Identification of EcpK, a bacterial tyrosine pseudokinase important for exopolysaccharide biosynthesis in *Myxococcus xanthus*

Luca Blöcher, Johannes Schwabe, Timo Glatter & Lotte Søgaard-Andersen

This file contains:

- Supplementary Figures 1-8
- Supplementary Tables 1-2
- Supplementary References

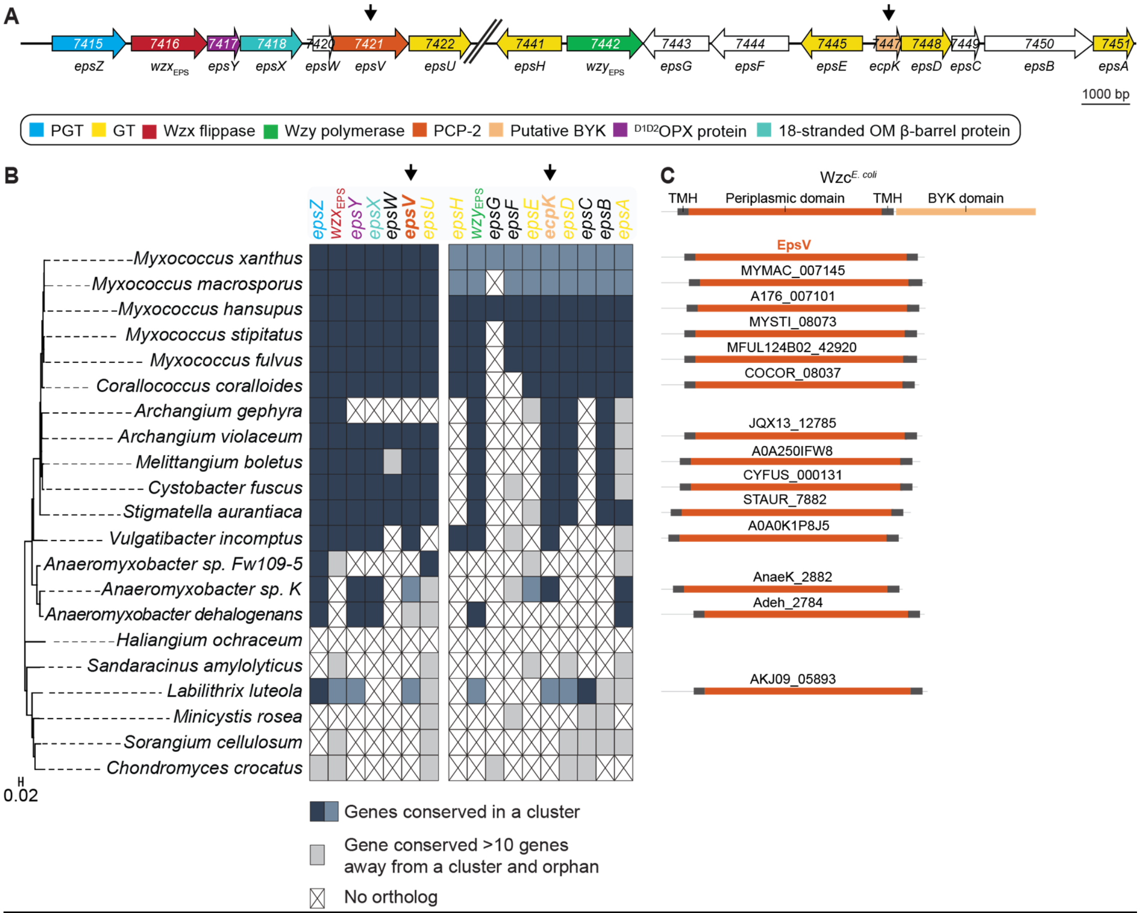

Figure S1. Myxobacterial gene clusters for EPS biosynthesis encode PCP-2 proteins lacking the BYK domain and orthologs of EcpK

(A) The two *eps* loci in *M. xanthus*. Gene names or MXAN_ numbers are indicated; genes are drawn to scale. The color code indicates the predicted functions of the gene products, shown in the lower panel, based on previous studies (1-5). EpsW is a single-domain response regulator required for EPS biosynthesis (6). *epsG* and *epsF* are annotated as encoding a magnesium transporter and a hybrid response regulator/histidine kinase, respectively. *epsF* is not important for EPS biosynthesis (5). The serine O-acetyltransferase EpsC is thought to participate in sugar nucleotide precursor biosynthesis but is not important for EPS biosynthesis (3-5). Similarly, EpsB, a predicted glycoside hydrolase, is not required for EPS biosynthesis (5).

(B) Left panel, a 16S rRNA-phylogenetic tree of fully sequenced Myxobacteria. Right panel, ortholog identification was performed using a reciprocal best hit BLASTP method. Genes within a distance of fewer than 10 genes were considered part of the same cluster, while clusters were considered distinct when separated by more than 10 genes. Genes within the same cluster are marked with the same color. Conserved orphan genes are colored light gray, and genes without orthologs are indicated by an X. In the upper panel, *M. xanthus* genes are color-coded according to the schematic in (A). Black arrows indicate genes encoding the EpsV ortholog (orange) and the EcpK ortholog (light orange) conserved across myxobacterial *eps* gene clusters.

(C) Conservation of domain structure of PCP-2 proteins encoded in myxobacterial *eps* gene clusters. Upper panel, domain organization of the prototypical PCP-2a Wzc$^{E. coli}$ (7). Transmembrane helices (TMH) are shown in dark grey, the periplasmic domain in orange,

and the BYK domain in light orange. Lower panel, domain structure of the PCP-2 EpsV and its myxobacterial orthologs, with domains colored according to their corresponding domains in Wzc$^{E\ coli}$.

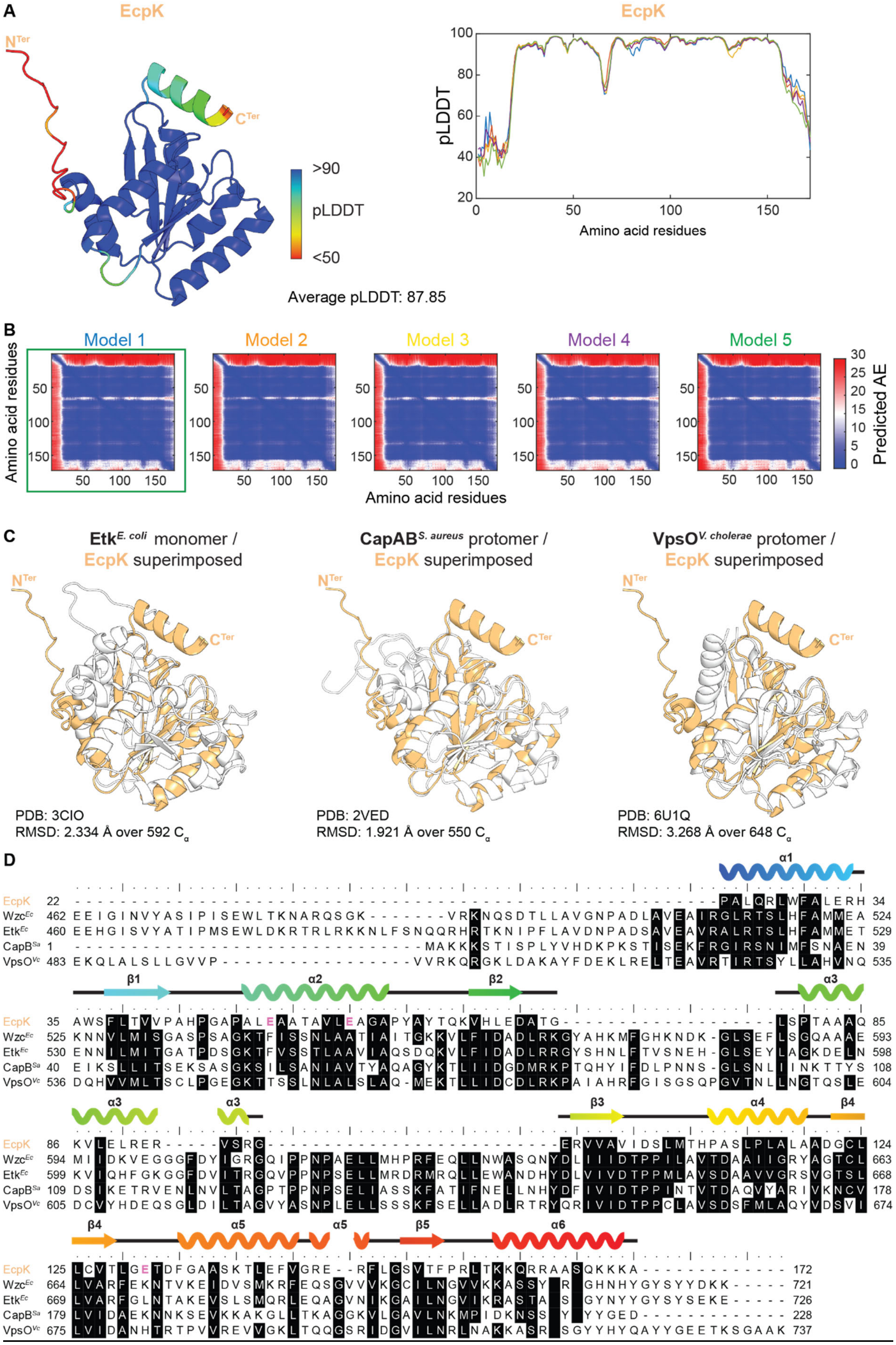

<u>Figure S2. AlphaFold2 model of EcpK.</u>

(A) Left panel, model rank 1 of EcpK colored according to pLDDT score, with average pLDDT score indicated. Right panel, pLDDT plot shown for the five generated models of EcpK.
(B) pAE plots shown for the five generated models of EcpK. Model rank 1 (highlighted by a green box) was selected for further analyses.
(C) Left panel, alignment of EcpK with Etk$^{E. coli}$ (8), with an RMSD of 2.334 over 592 C_α. Middle panel, alignment of EcpK with CapB$^{S. aureus}$ fused to the C-terminal extension of CapA$^{S. aureus}$ (9), with an RMSD of 1.921 over 550 C_α. Right panel, alignment of EcpK with VpsO$^{V. cholerae}$ (10), with an RMSD of 3.268 over 648 C_α.
(D) Sequence alignment of EcpK with the proteins identified in the Foldseek analysis in Fig. 2B. The secondary structure of EcpK based on the rank 1 AlphaFold2 model is shown above the sequence alignment, colored using a gradient from blue (N-terminus) to red (C-terminus) according to Fig. 2D. The BYK signature motifs (Walker A, Walker A', Walker B) and the Tyr-rich C-terminal tail are indicated. Consensus Walker motif sequences (11) are shown, with φ representing hydrophobic residues. Glu-residues E^{52}, E^{59} and E^{131} in EcpK are marked pink. Amino acid residue numbering corresponds to the full-length sequences of the proteins.

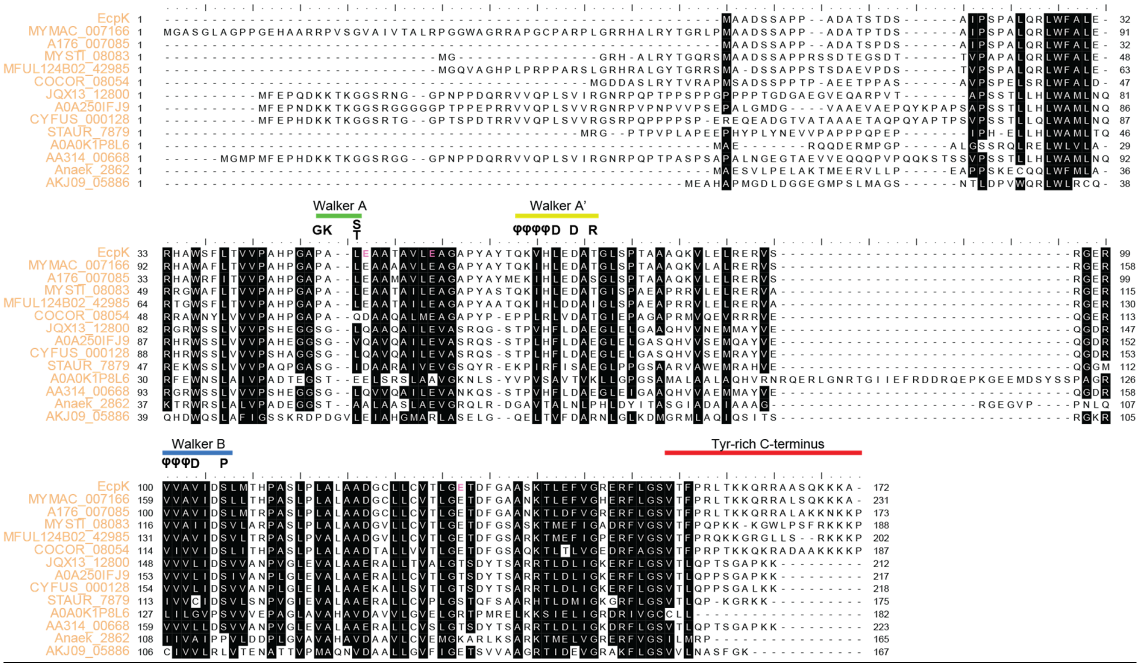

Figure S3. All myxobacterial EcpK orthologs are BY pseudokinases.

Sequence alignment of EcpK with its myxobacterial orthologs from Fig. S1B. The BYK signature motifs (Walker A, Walker A', Walker B) and the Tyr-rich C-terminal tail are indicated. Consensus Walker motif sequences (11) are shown, with φ representing hydrophobic residues. Glu-residues E^{52}, E^{59} and E^{131} in EcpK are marked pink. Amino acid residue numbering corresponds to the full-length sequences of the proteins.

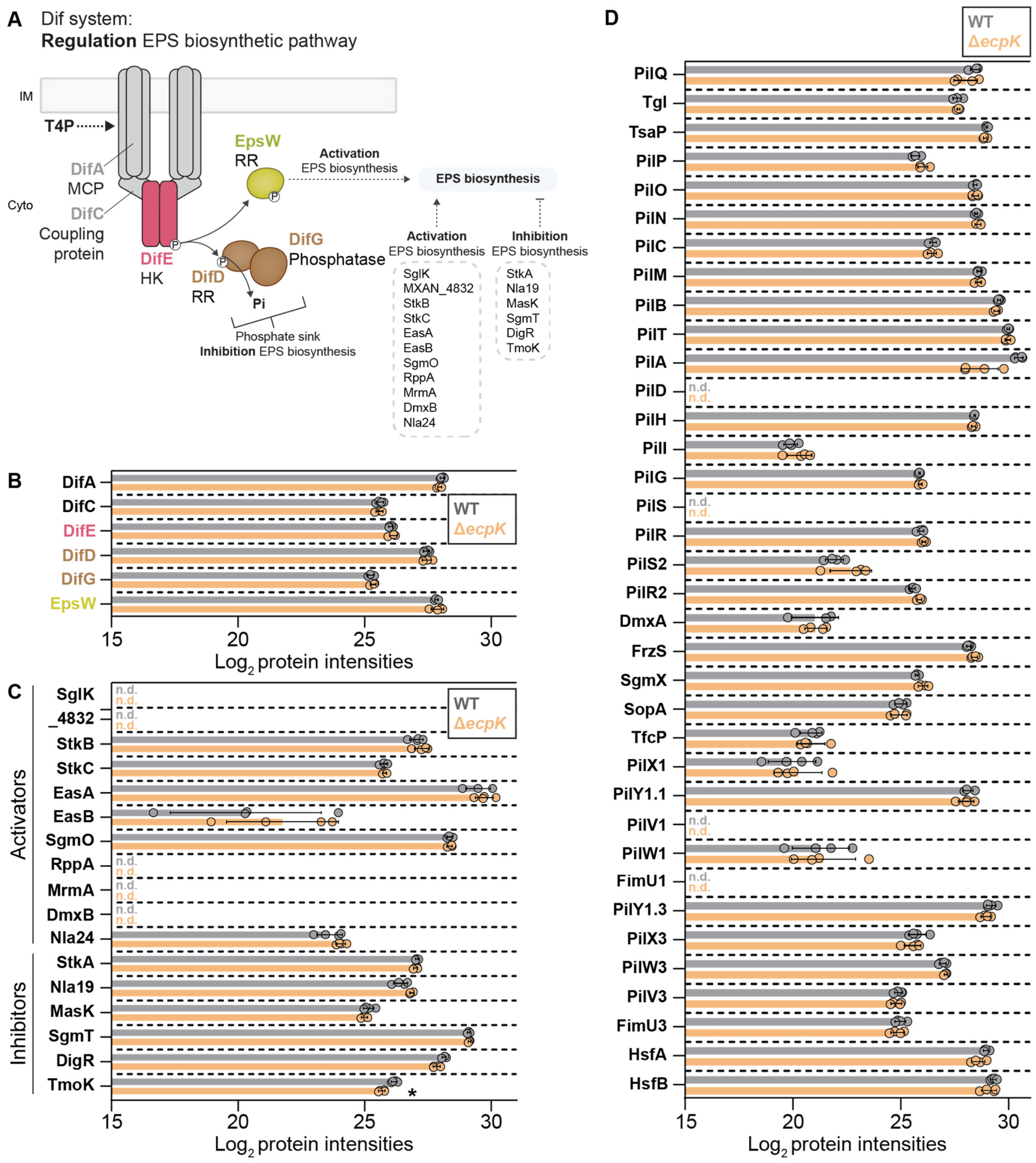

Figure S4. EcpK is not required for the accumulation of other proteins known to affect EPS biosynthesis.

(A) Schematic of the regulation of EPS biosynthesis in *M. xanthus* based on (12). Left panel, the Dif system activates EPS biosynthesis by an unknown mechanism *via* the phosphorylated response regulator EpsW, which is phosphorylated by DifE upon T4P extension (6, 13-19). DifD and DifG act as a phosphate sink, competing with EpsW for phosphorylation (17, 20). Right panel, list of additional regulators of EPS biosynthesis (12). Solid arrows indicate known mechanisms. Stippled arrows indicate unknown mechanisms. Abbreviations: MCP, methyl-accepting chemotaxis protein. HK, histidine kinase. RR, response regulator.

(B)-(D) Protein amounts in whole-cell proteomes of *M. xanthus* strains were quantified using LFQ mass spectrometry-based proteomics. Normalized Log_2 intensities of proteins in the indicated strains are shown. Each data point represents a biological replicate (n = 4 biological

replicates). Error bars indicate standard deviation across these replicates. *, $p < 0.01$, Welch's test against WT. "n.d." indicates that a protein was not detected in any replicate in a strain, and is shown in the corresponding strain's color. (B) Components of the Dif system, see (A) for details. (C) Additional proteins involved in regulating EPS biosynthesis (12). (D) Proteins necessary for the assembly of a functional T4P machine. PilQ is the multimeric OM secretin stabilized by LysM-domain protein TsaP (21-24). Tgl stimulates PilQ multimerization (23, 24). PilN/-O/-P are structural components in the periplasm. PilC/-M form the IM/cytoplasmic platform complex. PilB/-T are the extension and retraction ATPases, respectively, and PilA is the major pilin (22, 25). PilH/-I/-G are suggested to form an ABC transporter (26). PilD is the prepilin leader peptidase (26, 27). PilR/-S/-R2/-S2 are regulatory proteins (28, 29). DmxA is the diguanylate cyclase important for stimulating c-di-GMP synthesis during cytokinesis and incorporation of the T4P machine at the cell pole (30). FrzS, SgmX and SopA jointly regulate T4P formation (31-36). The Cluster_1 and Cluster_3 priming complexes facilitate T4P extension (37, 38), with TfcP stabilizing PilY1.1 (39). HsfA and HsfB are a phoshphorelay involved in regulating the transcription of, among other genes (40, 41), *cluster_1* and *cluster_3* (37).

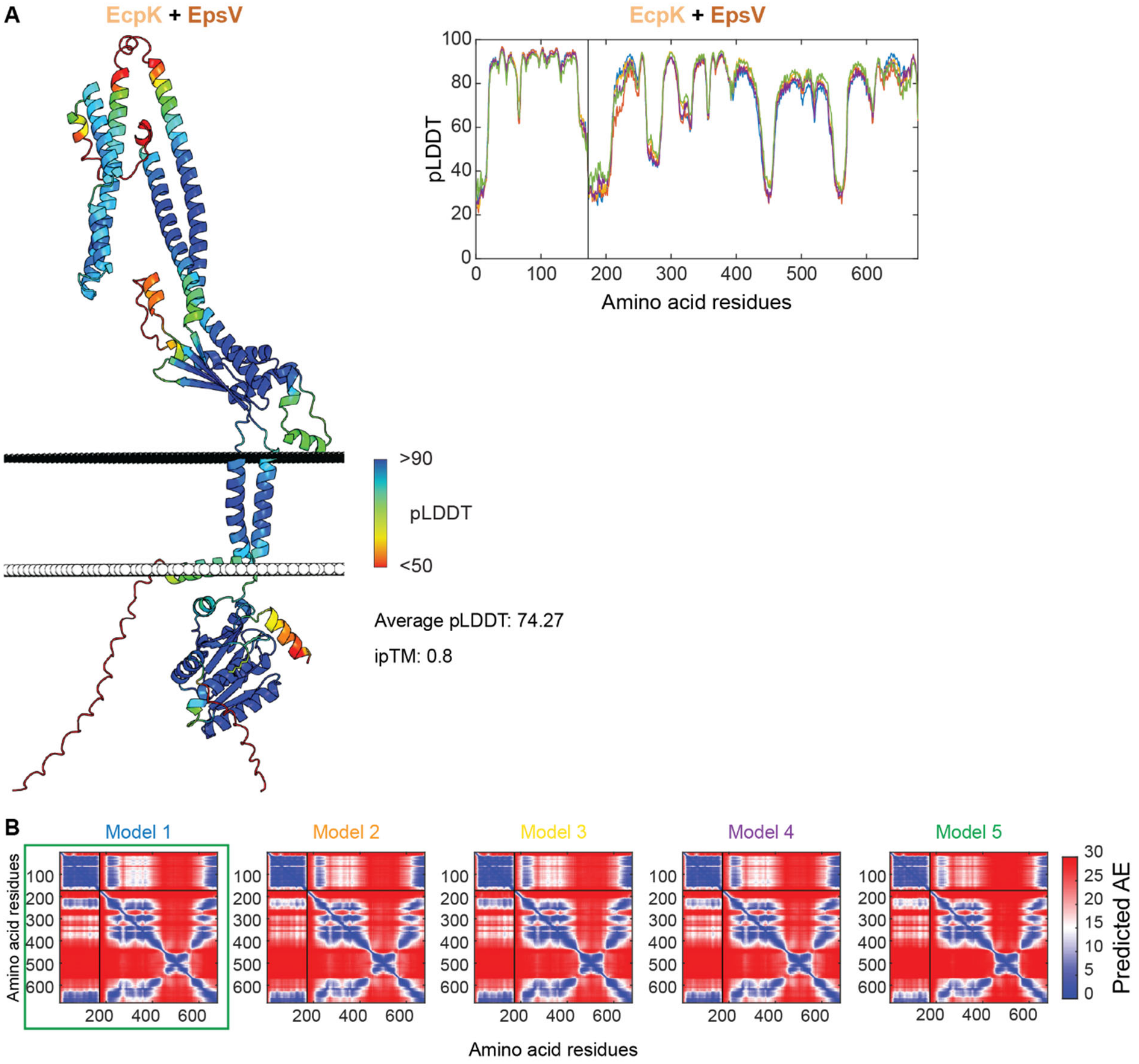

Figure S5. AlphaFold2-Multimer model of the EcpK/EpsV complex.

(A) Left panel, model rank 1 of the EcpK/EpsV complex colored according to pLDDT score, with average pLDDT and ipTM scores indicated. Right panel, pLDDT plot shown for the five generated models of the EcpK/EpsV complex.

(B) pAE plots shown for the five generated models of the EcpK/EpsV complex. Model rank 1 (highlighted by a green box) was selected for further analyses.

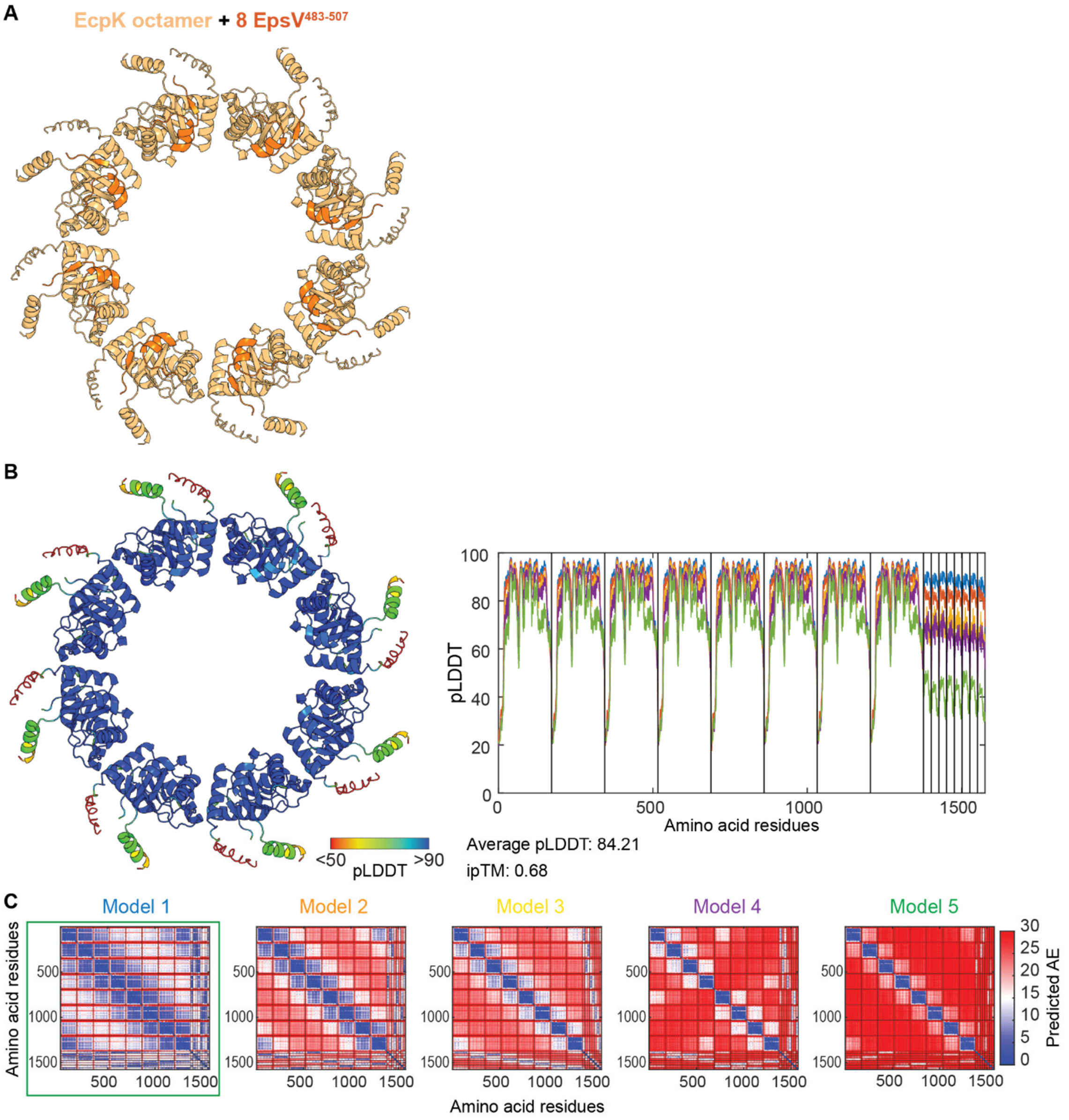

Figure S6. AlphaFold2-Multimer model of octameric EcpK/EpsV$^{483-507}$.

(A) Model rank 1 of octameric EcpK/EpsV$^{483-507}$ EcpK octamer colored in light orange, EpsV$^{483-507}$ colored in orange.

(B) Left panel, model rank 1 of octameric EcpK/EpsV$^{483-507}$ colored according to pLDDT score, with average pLDDT and ipTM scores indicated. Right panel, pLDDT plot shown for the five generated models of octameric EcpK/EpsV$^{483-507}$.

(C) pAE plots shown for the five generated models of octameric EcpK/EpsV$^{483-507}$. Model rank 1 (highlighted by a green box) was selected for further analysis.

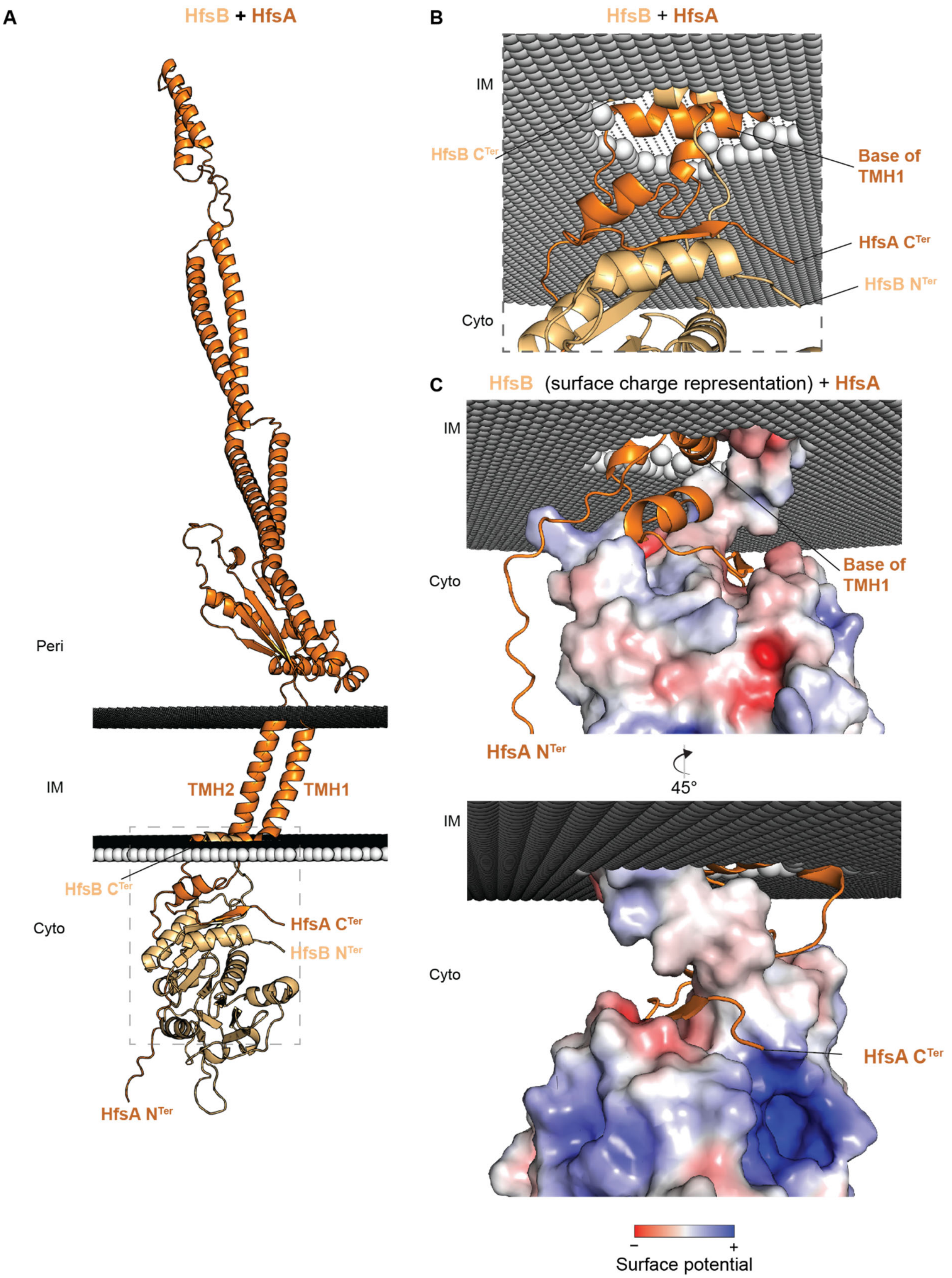

<u>Figure S7. Computational structural characterization of the HfsB-HfsA complex in *C. crescentus.*</u>

(A) AlphaFold2-Multimer model of the HfsB/HfsA complex. HfsB is shown in light orange, and HfsA in orange, with N- and C-termini highlighted. The predicted position of the HfsB/HfsA

complex within the membrane was calculated using the PPM server (42). Model rank 1 is shown.

(B) Zoomed in image of the HfsB/HfsA interaction highlighting that the last 27 C-terminal, cytoplasmic residues of HfsA interacts with HfsB. Note that HfsB's C-terminal α-helix is predicted to interact with the base of one of HfsA's two TMHs. N- and C-termini of the proteins are indicated.

(C) Zoomed in image of the HfsB/HfsA interaction with HfsB shown in the surface charge representation (contoured from +5 to −5 kT e^{-1}), which was computed using pdb2pqr *via* the Adaptive Poisson-Boltzmann Solver server (43). Negative and positive charges are colored red and blue, respectively. Model rank 1 of the HfsA/HfsB complex, with HfsA in orange and HfsB in light-orange, respectively. N- and C-termini of HfsA are indicated.

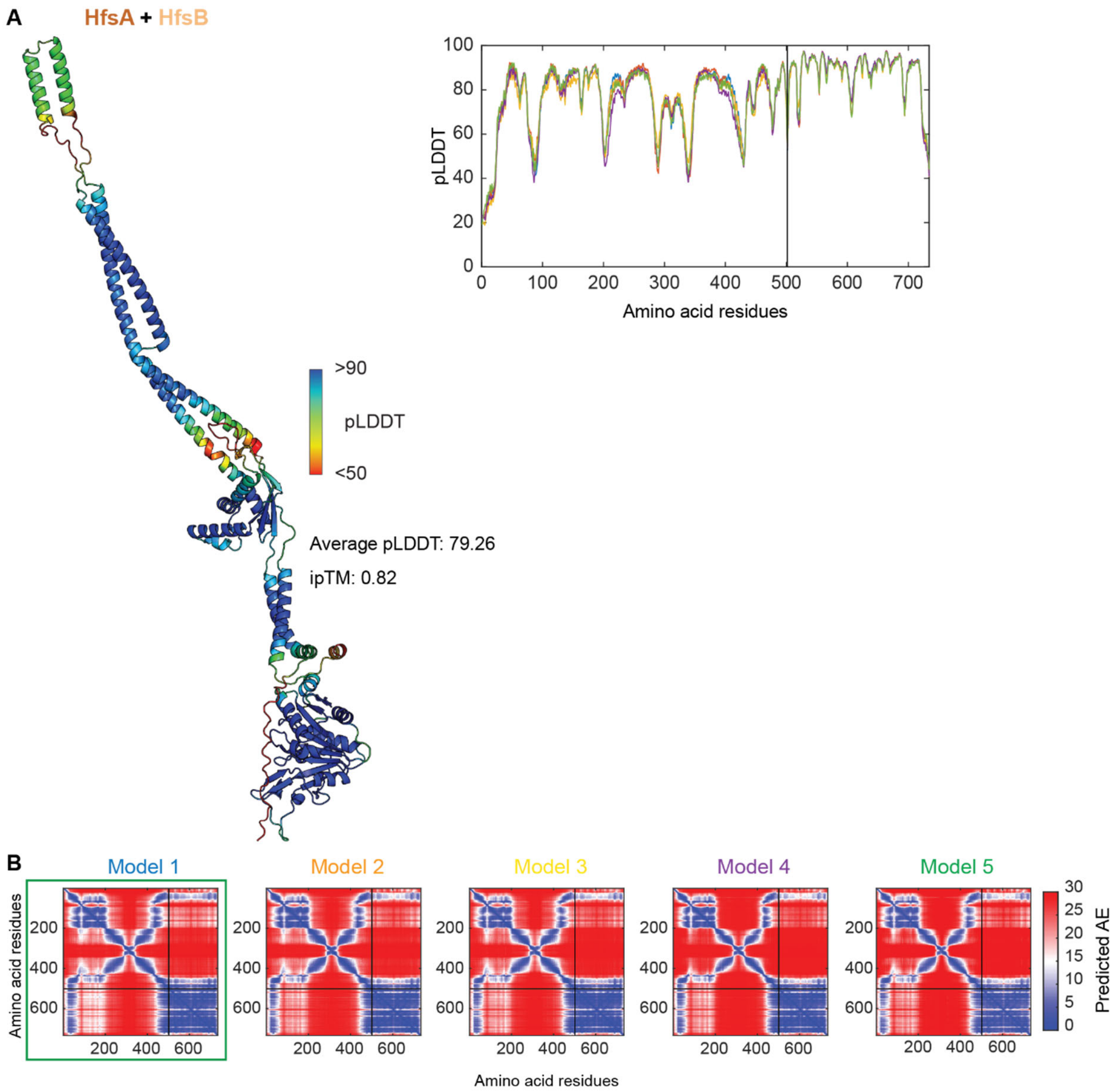

<u>Figure S8. AlphaFold2-Multimer model of the HfsA-HfsB complex.</u>

(A) Left panel, model rank 1 of the HfsA/HfsB complex colored according to pLDDT score, with average pLDDT and ipTM scores indicated. Right panel, pLDDT plot shown for the five generated models of the HfsA/HfsB complex.

(B) pAE plots shown for the five generated models of the HfsA/HfsB complex. Model rank 1 (highlighted by a green box) was selected for further analyses.

Table S1. Oligonucleotides used in this work[1]

Primer name	Sequence 5'-3'	Brief description
LB1	ACTGGGTACCGCTCCACGCCCATGGGTG	For Δ*ecpK*
LB40	GGACGCCGCGGAATCGGCTGCCATGGG	For Δ*ecpK*
LB18	AGCCGATTCCGCGGCGTCCCAGAAGAAG	For Δ*ecpK*
LB4	ACTGTCTAGAGGGTCAGGGGGTTGGTCT	For Δ*ecpK*
LB5	AGCACCTTGCGGTACGTG	For Δ*ecpK*
LB6	GCCGCGCAGATACTCCAT	For Δ*ecpK*
LB7	TGCAACGGCTGTGGTTCG	For Δ*ecpK*
LB8	TGACGCTGCCGAGGAAGC	For Δ*ecpK*
LB9	ACTGAAGCTTTCATGCCTTCTTCTTCTGGGACGCC	For complementation of Δ*ecpK*
LB10	ACTGTCTAGAAGGCCAGGCACCGGG	For complementation of Δ*ecpK*
tuf2_q3-for	AGTGGAAGTCGTTGGTCTGC	Reference gene RT-qPCR
tuf2_q3-rev	TTGGTGTGCGGGGTGATG	Reference gene RT-qPCR
LB35	AGAAGGTCCACCTGGAAGAC	RT-qPCR for *ecpK*
LB36	ATCAACGAGTCGATGACGG	RT-qPCR for *ecpK*
7421-q-2-for	CGACGCGGTCTTCTTTTTGA	RT-qPCR for *epsV*
7421-q-2-rev	CATGATTTTGCTGACGCCCA	RT-qPCR for *epsV*
LB59	ACTGAAGCTTGGCAGCCGATTCCAGCGC	BACTH with EcpK and EpsV
LB60	ACTGTCTAGAGGCAGCCGATTCCAGCGC	BACTH with EcpK and EpsV
LB61	ACTGAAGCTTAACGGTCCCCGCGCCCGGG	BACTH with EcpK and EpsV
LB62	ACTGGGTACCGCCTTCTTCTTCTGGGAC	BACTH with EcpK and EpsV
LB63	ACTGTCTAGAAACGGTCCCCGCGCCCGGGGCTC	BACTH with EcpK and EpsV
LB64	ACTGGGTACCCCGCGCCGCTCCAGCTCCGC	BACTH with EcpK and EpsV
LB65	ACTGAGATCTAGAGTGCGCCGCTCCAGCTCCGCCAG	BACTH with EcpK and EpsV

[1] Underlined sequences indicate restriction sites.

Table S2. Fully sequenced myxobacterial genomes used for the 16S RNA tree

Species and strain name
Anaeromyxobacter dehalogenans 2CP-C
Anaeromyxobacter sp. Fw109-5
Anaeromyxobacter sp. K
Archangium gephyra DSM 2261
Archangium violaceum Cb SDU34
Chondromyces crocatus Cm c5
Corallococcus coralloides DSM 2259
Cystobacter fuscus DSM 52655
Haliangium ochraceum DSM 14365
Labilithrix luteola DSM 27648
Melittangium boletus DSM 14713SG
Minicystis rosea DSM 24000
Myxococcus macrosporus DSM 14675
Myxococcus hansupus (*Myxococcus* sp. mixupus)
Myxococcus stipitatus DSM 14675
Myxococcus xanthus DK1622
Sandaracinus amylolyticus DSM 53668
Sorangium cellulosum So ce 56
Stigmatella aurantiaca DW4/3-1
Vulgatibacter incomptus DSM 27710

Supplementary references

1. Schwabe J, Pérez-Burgos M, Herfurth M, Glatter T, Søgaard-Andersen L. 2022. Evidence for a widespread third system for bacterial polysaccharide export across the outer membrane comprising a composite OPX/β-barrel translocon. mBio 13:e0203222.

2. Saïdi F, Mahanta U, Panda A, Kezzo AA, Jolivet NY, Bitazar R, John G, Martinez M, Mellouk A, Calmettes C, Chang YW, Sharma G, Islam ST. 2022. Bacterial outer membrane polysaccharide export (OPX) proteins occupy three structural classes with selective β-barrel porin requirements for polymer secretion. Microbiol Spectr 10:e0129022.

3. Islam ST, Vergara Alvarez I, Saïdi F, Guiseppi A, Vinogradov E, Sharma G, Espinosa L, Morrone C, Brasseur G, Guillemot JF, Benarouche A, Bridot JL, Ravicoularamin G, Cagna A, Gauthier C, Singer M, Fierobe HP, Mignot T, Mauriello EMF. 2020. Modulation of bacterial multicellularity via spatio-specific polysaccharide secretion. PLoS Biol 18:e3000728.

4. Pérez-Burgos M, Garcia-Romero I, Jung J, Schander E, Valvano MA, Søgaard-Andersen L. 2020. Characterization of the exopolysaccharide biosynthesis pathway in *Myxococcus xanthus*. J Bacteriol 202:e00335-20.

5. Lu A, Cho K, Black WP, Duan XY, Lux R, Yang Z, Kaplan HB, Zusman DR, Shi W. 2005. Exopolysaccharide biosynthesis genes required for social motility in *Myxococcus xanthus*. Mol Microbiol 55:206-20.

6. Black WP, Wang L, Davis MY, Yang Z. 2015. The orphan response regulator EpsW is a substrate of the DifE kinase and it regulates exopolysaccharide in *Myxococcus xanthus*. Sci Rep 5:17831.

7. Yang Y, Liu J, Clarke BR, Seidel L, Bolla JR, Ward PN, Zhang P, Robinson CV, Whitfield C, Naismith JH. 2021. The molecular basis of regulation of bacterial capsule assembly by Wzc. Nat Commun 12:4349.

8. Lee DC, Zheng J, She YM, Jia Z. 2008. Structure of *Escherichia coli* tyrosine kinase Etk reveals a novel activation mechanism. EMBO J 27:1758-66.

9. Olivares-Illana V, Meyer P, Bechet E, Gueguen-Chaignon V, Soulat D, Lazereg-Riquier S, Mijakovic I, Deutscher J, Cozzone AJ, Laprevote O, Morera S, Grangeasse C, Nessler S. 2008. Structural basis for the regulation mechanism of the tyrosine kinase CapB from *Staphylococcus aureus*. PLoS Biol 6:e143.

10. Schwechheimer C, Hebert K, Tripathi S, Singh PK, Floyd KA, Brown ER, Porcella ME, Osorio J, Kiblen JTM, Pagliai FA, Drescher K, Rubin SM, Yildiz FH. 2020. A tyrosine phosphoregulatory system controls exopolysaccharide biosynthesis and biofilm formation in *Vibrio cholerae*. Plos Pathogens 16:e1008745.

11. Grangeasse C, Nessler S, Mijakovic I. 2012. Bacterial tyrosine kinases: evolution, biological function and structural insights. Philos Trans R Soc Lond B Biol Sci 367:2640-55.

12. Pérez-Burgos M, Søgaard-Andersen L. 2020. Biosynthesis and function of cell-surface polysaccharides in the social bacterium *Myxococcus xanthus*. Biol Chem 401:1375-1387.

13. Bellenger K, Ma X, Shi W, Yang Z. 2002. A CheW homologue is required for *Myxococcus xanthus* fruiting body development, social gliding motility, and fibril biogenesis. J Bacteriol 184:5654-60.

14. Yang ZM, Geng YZ, Xu D, Kaplan HB, Shi WY. 1998. A new set of chemotaxis homologues is essential for *Myxococcus xanthus* social motility. Mol Microbiol 30:1123-1130.

15. Moak PL, Black WP, Wallace RA, Li Z, Yang Z. 2015. The Hsp70-like StkA functions between T4P and Dif signaling proteins as a negative regulator of exopolysaccharide in *Myxococcus xanthus*. PeerJ 3:e747.

16. Xu Q, Black WP, Nascimi HM, Yang Z. 2011. DifA, a methyl-accepting chemoreceptor protein-like sensory protein, uses a novel signaling mechanism to regulate exopolysaccharide production in *Myxococcus xanthus*. J Bacteriol 193:759-67.

17. Black WP, Schubot FD, Li Z, Yang ZM. 2010. Phosphorylation and dephosphorylation among Dif Chemosensory proteins essential for exopolysaccharide regulation in *Myxococcus xanthus*. J Bacteriol 192:4267-4274.

18. Black WP, Xu Q, Yang Z. 2006. Type IV pili function upstream of the Dif chemotaxis pathway in *Myxococcus xanthus* EPS regulation. Mol Microbiol 61:447-56.

19. Yang Z, Ma X, Tong L, Kaplan HB, Shimkets LJ, Shi W. 2000. *Myxococcus xanthus dif* genes are required for biogenesis of cell surface fibrils essential for social gliding motility. J Bacteriol 182:5793-8.

20. Black WP, Yang Z. 2004. *Myxococcus xanthus* chemotaxis homologs DifD and DifG negatively regulate fibril polysaccharide production. J Bacteriol 186:1001-8.

21. Siewering K, Jain S, Friedrich C, Webber-Birungi MT, Semchonok DA, Binzen I, Wagner A, Huntley S, Kahnt J, Klingl A, Boekema EJ, Søgaard-Andersen L, van der Does C. 2014. Peptidoglycan-binding protein TsaP functions in surface assembly of type IV pili. Proc Natl Acad Sci U S A 111:E953-61.

22. Chang YW, Rettberg LA, Treuner-Lange A, Iwasa J, Søgaard-Andersen L, Jensen GJ. 2016. Architecture of the type IVa pilus machine. Science 351:aad2001.

23. Herfurth M, Pérez-Burgos M, Søgaard-Andersen L. 2023. The mechanism for polar localization of the type IVa pilus machine in *Myxococcus xanthus*. mBio:e0159323.

24. Wall D, Kolenbrander PE, Kaiser D. 1999. The *Myxococcus xanthus pilQ* (*sglA*) gene encodes a secretin homolog required for type IV pilus biogenesis, social motility, and development. J Bacteriol 181:24-33.

25. Jakovljevic V, Leonardy S, Hoppert M, Søgaard-Andersen L. 2008. PilB and PilT are ATPases acting antagonistically in type IV pilus function in *Myxococcus xanthus*. J Bacteriol 190:2411-21.

26. Wu SS, Wu J, Cheng YL, Kaiser D. 1998. The *pilH* gene encodes an ABC transporter homologue required for type IV pilus biogenesis and social gliding motility in *Myxococcus xanthus*. Mol Microbiol 29:1249-61.

27. Nunn DN, Lory S. 1991. Product of the *Pseudomonas aeruginosa* gene *pilD* is a prepilin leader peptidase. Proc Natl Acad Sci U S A 88:3281-5.

28. Bretl DJ, Muller S, Ladd KM, Atkinson SN, Kirby JR. 2016. Type IV-pili dependent motility is co-regulated by PilSR and PilS2R2 two-component systems via distinct pathways in *Myxococcus xanthus*. Mol Microbiol 102:37-53.

29. Wu SS, Kaiser D. 1997. Regulation of expression of the *pilA* gene in *Myxococcus xanthus*. J Bacteriol 179:7748-58.

30. Perez-Burgos M, Herfurth M, Kaczmarczyk A, Harms A, Huber K, Jenal U, Glatter T, Søgaard-Andersen L. 2024. A deterministic, c-di-GMP-dependent program ensures the generation of phenotypically similar, symmetric daughter cells during cytokinesis. Nat Commun 15:6014.

31. Oklitschek M, Carreira LAM, Muratoglu M, Søgaard-Andersen L, Treuner-Lange A. 2024. Combinatorial control of type IVa pili formation by the four polarized regulators MglA, SgmX, FrzS, and SopA. J Bacteriol 206:e00108-24.

32. Fraser JS, Merlie JP, Jr., Echols N, Weisfield SR, Mignot T, Wemmer DE, Zusman DR, Alber T. 2007. An atypical receiver domain controls the dynamic polar localization of the *Myxococcus xanthus* social motility protein FrzS. Mol Microbiol 65:319-32.

33. Ward MJ, Lew H, Zusman DR. 2000. Social motility in *Myxococcus xanthus* requires FrzS, a protein with an extensive coiled-coil domain. Mol Microbiol 37:1357-71.

34. Bautista S, Schmidt V, Guiseppi A, Mauriello EMF, Attia B, Elantak L, Mignot T, Mercier R. 2023. FrzS acts as a polar beacon to recruit SgmX, a central activator of type IV pili during *Myxococcus xanthus* motility. EMBO J 42:e111661.

35. Potapova A, Carreíra LAM, Søgaard-Andersen L. 2020. The small GTPase MglA together with the TPR domain protein SgmX stimulates type IV pili formation in *M. xanthus*. Proc Natl Acad Sci U S A 117:23859-23868.

36. Mercier R, Bautista S, Delannoy M, Gibert M, Guiseppi A, Herrou J, Mauriello EMF, Mignot T. 2020. The polar Ras-like GTPase MglA activates type IV pilus via SgmX to enable twitching motility in *Myxococcus xanthus*. Proc Natl Acad Sci U S A 117:28366-28373.

37. Xue S, Mercier R, Guiseppi A, Kosta A, De Cegli R, Gagnot S, Mignot T, Mauriello EMF. 2022. The differential expression of PilY1 proteins by the HsfBA phosphorelay allows twitching motility in the absence of exopolysaccharides. PLoS Genet 18:e1010188.

38. Treuner-Lange A, Chang YW, Glatter T, Herfurth M, Lindow S, Chreifi G, Jensen GJ, Søgaard-Andersen L. 2020. PilY1 and minor pilins form a complex priming the type IVa pilus in *Myxococcus xanthus*. Nat Commun 11:5054.

39. Herfurth M, Treuner-Lange A, Glatter T, Wittmaack N, Hoiczyk E, Pierik AJ, Søgaard-Andersen L. 2022. A noncanonical cytochrome c stimulates calcium binding by PilY1 for type IVa pili formation. Proc Natl Acad Sci U S A 119:e2115061119.

40. Volz C, Kegler C, Muller R. 2012. Enhancer binding proteins act as hetero-oligomers and link secondary metabolite production to myxococcal development, motility, and predation. Chem Biol 19:1447-59.

41. Ueki T, Inouye S. 2002. Transcriptional activation of a heat-shock gene, lonD, of *Myxococcus xanthus* by a two component histidine-aspartate phosphorelay system. J Biol Chem 277:6170-7.

42. Lomize AL, Todd SC, Pogozheva ID. 2022. Spatial arrangement of proteins in planar and curved membranes by PPM 3.0. Protein Science 31:209-220.

43. Jurrus E, Engel D, Star K, Monson K, Brandi J, Felberg LE, Brookes DH, Wilson L, Chen J, Liles K, Chun M, Li P, Gohara DW, Dolinsky T, Konecny R, Koes DR, Nielsen JE, Head-Gordon T, Geng W, Krasny R, Wei GW, Holst MJ, McCammon JA, Baker NA. 2018. Improvements to the APBS biomolecular solvation software suite. Protein Sci 27:112-128.
